# The innovative model based on artificial intelligence algorithms to predict recurrence risk of patients with postoperative breast cancer

**DOI:** 10.3389/fonc.2023.1117420

**Published:** 2023-03-07

**Authors:** Lixuan Zeng, Lei Liu, Dongxin Chen, Henghui Lu, Yang Xue, Hongjie Bi, Weiwei Yang

**Affiliations:** ^1^ Department of Pathology, Harbin Medical University, Harbin, China; ^2^ Department of Breast Surgery, The Third Affiliated Hospital of Harbin Medical University, Harbin, China; ^3^ Department of Dermatology, The Second Affiliated Hospital of Harbin Medical University, Harbin, China

**Keywords:** breast cancer, recurrence risk, LSTM, XGBoost, SVM

## Abstract

**Purpose:**

This study aimed to develop a machine learning model to retrospectively study and predict the recurrence risk of breast cancer patients after surgery by extracting the clinicopathological features of tumors from unstructured clinical electronic health record (EHR) data.

**Methods:**

This retrospective cohort included 1,841 breast cancer patients who underwent surgical treatment. To extract the principal features associated with recurrence risk, the clinical notes and histopathology reports of patients were collected and feature engineering was used. Predictive models were next conducted based on this important information. All algorithms were implemented using Python software. The accuracy of prediction models was further verified in the test cohort. The area under the curve (AUC), precision, recall, and F1 score were adopted to evaluate the performance of each model.

**Results:**

A training cohort with 1,289 patients and a test cohort with 552 patients were recruited. From 2011 to 2019, a total of 1,841 textual reports were included. For the prediction of recurrence risk, both LSTM, XGBoost, and SVM had favorable accuracies of 0.89, 0.86, and 0.78. The AUC values of the micro-average ROC curve corresponding to LSTM, XGBoost, and SVM were 0.98 ± 0.01, 0.97 ± 0.03, and 0.92 ± 0.06. Especially the LSTM model achieved superior execution than other models. The accuracy, F1 score, macro-avg F1 score (0.87), and weighted-avg F1 score (0.89) of the LSTM model produced higher values. All *P* values were statistically significant. Patients in the high-risk group predicted by our model performed more resistant to DNA damage and microtubule targeting drugs than those in the intermediate-risk group. The predicted low-risk patients were not statistically significant compared with intermediate- or high-risk patients due to the small sample size (188 low-risk patients were predicted *via* our model, and only two of them were administered chemotherapy alone after surgery). The prognosis of patients predicted by our model was consistent with the actual follow-up records.

**Conclusions:**

The constructed model accurately predicted the recurrence risk of breast cancer patients from EHR data and certainly evaluated the chemoresistance and prognosis of patients. Therefore, our model can help clinicians to formulate the individualized management of breast cancer patients.

## Introduction

According to estimates from the Global Cancer Observatory (GLOBOCAN) in 2020, the incidence of female breast cancer ranked first, surpassing even lung cancer (1). Meanwhile, in China, the incidence of breast cancer has risen to the fourth among all cancer types and shows a trend of younger age (2). Breast cancer seriously harms women’s life and health. Accurately evaluating the recurrence risk of postoperative breast cancer patients can greatly improve their prognosis through appropriate treatment (3).

With the digitization of medical information, machine learning models have been applied in oncology (4–6). In 2021, artificial intelligence (AI) was used to predict the occurrence of breast cancer metastasis by learning from clinical electronic health record (EHR) data to support individualized diagnosis for patients (7). EHRs contain numerous longitudinal records, including histopathology, molecular markers related to breast cancer, radiology, and clinical information. However, the manual integration of prognostic information from EHRs by clinical experts is time-consuming, laborious, and costly (8, 9). Therefore, precisely assessing the recurrence risk and improving the efficiency of clinical evaluation plays a crucial role in controlling the disease burden of breast cancer.

Support vector machine (SVM) is a powerful learning algorithm that is capable of addressing various dimensions of data through different kernel functions. For example, breast cancer cells were classified *in vitro* with an accuracy of 93% using linear and radial basis function (RBF) kernel SVMs (10). Extreme gradient boosting (XGBoost) is a decision tree-based algorithm that is widely used in machine learning. It minimizes the loss function of the model through a gradient descent algorithm and implements the speed and performance of gradient-boosted decision trees (11). Furthermore, artificial neural networks (ANN) comprise a fundamental component of deep learning algorithms, demonstrating great potential in building high prediction accuracy (12–15). Currently, AI algorithms have proven successful in processing clinical image data, obtaining desired prediction results (16–18). For example, a two-stage convolutional neural network (CNN) model was proposed to predict the occurrence of myocardial infarction and localize the site of infarction based on vectorcardiogram signals (19). However, further research is needed to process clinical non-image data using machine learning.

In this study, we aimed to develop an artificial intelligence prediction model to regressively identify the recurrence risk of breast cancer patients after operation. We used SVM, XGBoost, and LSTM algorithms to integrate the histopathological and molecular characteristics of tumors in patients’ EHRs. We also validated the model’s performance in predicting risk categories for patients who received neoadjuvant and postoperative chemotherapy or postoperative chemotherapy alone, which can provide a precise assessment for personalized medicine for cancer patients. Our study made the following important contributions:

- Developed models based on three AI algorithms (SVM, XGBoost, and LSTM) that accurately predicted the recurrence risk of postoperative breast cancer patients.
- Provided a suitable model for recurrence risk prediction that reflects the chemotherapy resistance of postoperative patients.
- Our LSTM model approximately evaluated the actual benefit of patients receiving neoadjuvant chemotherapy.
- Predicted recurrence risk by the LSTM model, accurately reflecting the prognosis of postoperative breast cancer patients.

## Methods

### Clinicopathological data of breast cancer patients

**Figure 1:**
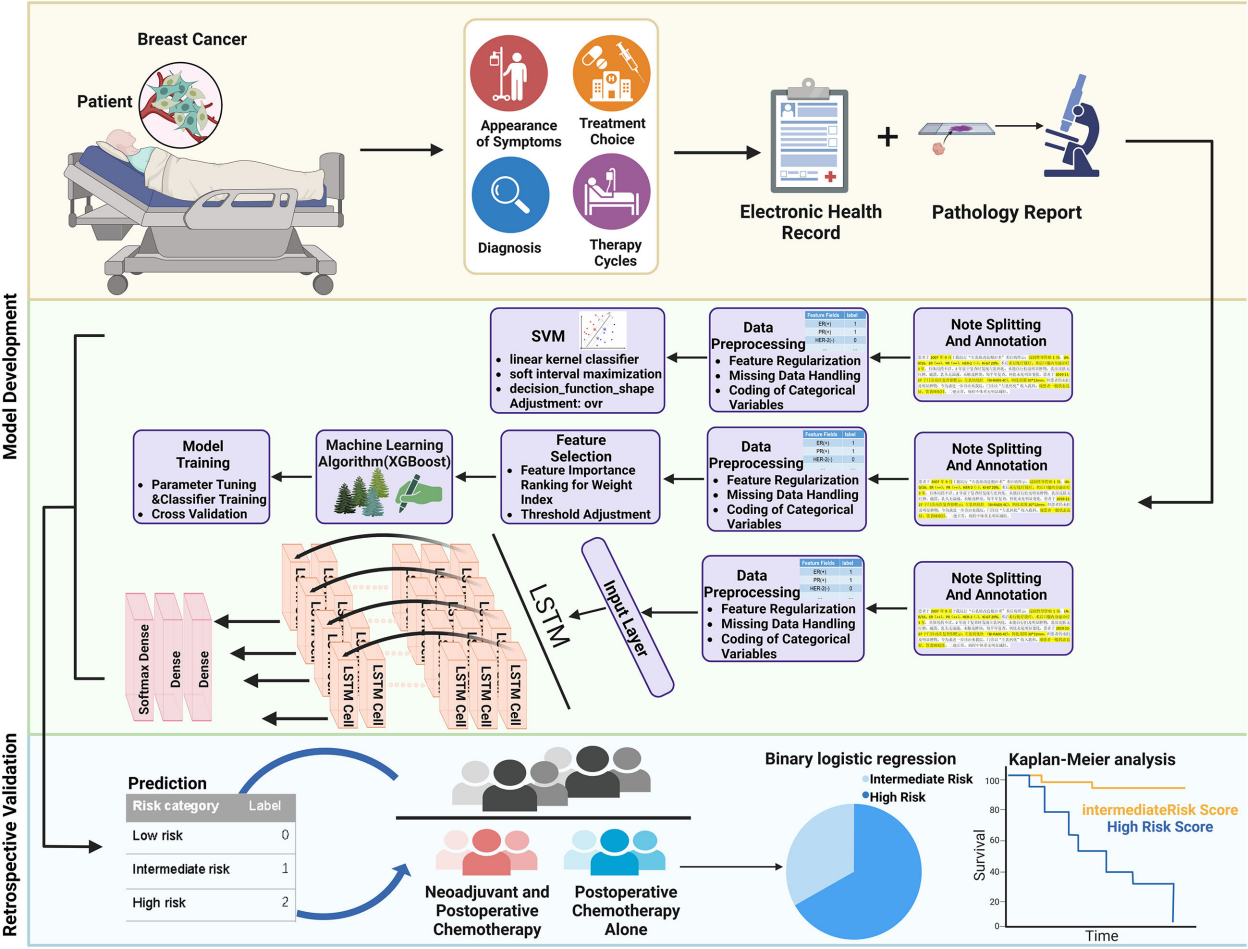
Overall workflow of the study. Histopathological features of breast cancer were first extracted and annotated by retrospective retrieving EHR data of breast cancer patients. The preprocessed information was next generated as a feature set, and models were trained to predict the recurrence risk of patients. The model was further validated in patients who received neoadjuvant and postoperative chemotherapy or postoperative chemotherapy alone.

This retrospective study was designed to predict the risk of breast cancer patients who underwent surgery through automated models. The overall methodology of this study is illustrated in **Figure 1**. A total of 1,962 patients with breast cancer were recruited from the Third Affiliated Hospital of Harbin Medical University from 11/05/2011, to 29/12/2019. There were 121 (6.1%) patients initially excluded because of incomplete pathological examination results or lack of clinical notes. Eventually, 1,841 patients were included in this retrospective analysis. A total of 432 patients underwent different treatment regimens following surgery and had complete treatment information, including radiotherapy, chemotherapy alone, combination therapy, endocrine therapy, and targeted therapy. Completed follow-up information of postoperative patients was collected, containing the surveillance of contralateral breast cancer, lymph node metastases, distant organ metastases, and other relevant monitoring. All study procedures were thoroughly reviewed and received ethical approval from the Harbin Medical University Ethics Committee. Informed written consent was obtained from each participant prior to their involvement in the study. A detailed description of the patient characteristics is found in **Supplemental Table 1**.

### Data parsing and feature extraction

Data preprocessing plays an important role in the application of machine learning (20). Since medical professionals have multiple expressions in medical reports, we first broke each note into blocks and standardized the reporting format, mainly regarding its clinical concepts and attributes. More details are explained in the **Supplement Data**. We further used natural language processing (NLP) based on the regular expression (regex) in Python to extract all key terms from EHRs (21). The regular expression can quickly analyze large volumes of textual information and has a specialized syntax. We compiled the regular expression pattern for each feature according to this specified syntax, thus accurately matching specific strings (22). An example below shows the feature extraction process:

re.compile (r’ER\([\+\-].*?\)|ER\([\+\-].*?\)’,re.I)

- re.compile: this regular expression was given to return every line in which the term “ER(+)” or “ER(-)” is present. Parentheses were probably performed with Chinese and English format in our data.
- re.I: re.IGNORECASE, this function was given to return values treated as case-insensitive.
- re.findall() function was next given to return all the matched strings “[-]” or “[+]” in the form of a list of numeric labels “0” or “1.”

In addition, NegEx was used to identify whether a term had been negated, effectively rectifying false-positive cases (23). For instance, “lymph nodes are not enlarged,” “lymph node-negative,” and “no evidence of lymphovascular invasion” were considered negative. After feature extraction, we combined all the features and created a dataset. The output values of all samples were displayed on the label with “=1” to match successfully; else, it was “=0” (**Table 1**). The missing values in our raw data were filled in “=0.” Eventually, the accuracy of feature extraction was estimated using the actual values in the original text snippets (24). Correct extraction was considered true positive (TP) when the extracted values matched the actual values. A classification for the module was regarded as false positive (FP) when the extracted values did not match the actual values. Missed entities were considered false negative (FN) when actual values were available, but no extracted values were reported. It was regarded as a true negative (TN) when no extracted values were produced and there were no actual values. **Supplementary Table 2** shows the confusion matrix for evaluated extraction.

**Table 1:** Feature extracted labels and descriptions.

Feature names	Feature descriptions	Illustrative example
Patients	Patient ID	616402
Age	Years	51
Menopausal status	Pre = 0 Post = 1	1
ER	Estrogen receptor-positive = 1 Estrogen receptor-negative = 0	1
PR	Progesterone receptor-positive = 1 Progesterone receptor-negative = 0	1
HER2	HER2/neu gene overexpressed or amplified = 1 HER2/neu gene neither overexpressed nor amplified = 0	0
Tumor size	Pathological tumor size ≤2cm = 0 Pathological tumor size >2 cm = 1	0
LNM	Positive lymph node metastasis = 1 Negative lymph node metastasis = 0	0
Number of LNM	The number of lymph node metastases	
G1	Pathology grade I = 1 Pathology grade II, pathology grade III = 0	1
G2	Pathology grade II = 1 Pathology grade I, pathology grade III = 0	0
G3	Pathology grade III = 1 Pathology grade I, pathology grade II = 0	0
LVI	Lympho-vascular invasion (+) = 1 Lympho-vascular invasion (-) = 0	0
Ki-67 (%)	The median pathology of Ki-67 proliferative index	5
Distant organ metastasis	Distant organ metastasis = 1 Non-distant organ metastasis = 0	0
Label	Low risk = 0 Intermediate risk = 1 High risk = 2	0

### Model prediction and evaluation

The recurrence risk of postoperative breast cancer patients was according to the clinical guidelines for the diagnosis and treatment of Breast cancer in 2021 of Chinese Anti-Cancer Association, Committee of Breast Cancer Society (CACA-CBCS) (**Supplemental Table 3**) (25). It has performed an important premise that Chinese clinicians base on to comprehensively assess and formulate treatment regimens.

Each prediction model was implemented through the Scikit-learn library in Python. First, the dataset was loaded into the Pandas dataframe and split into a training set (70%) and a test (30%) set with the train_test_split function. In order to avoid extreme values, the fillna() function was executed to fill the vacant values with default values and scale numerical variables for range adjustment.

SVM is a supervised learning algorithm commonly employed in binary classification and regression problems. The basic principle of SVM is to identify a decision boundary so that samples can be separated from different classes (26). In this study, the sklearn.svm.SVC function was adopted to solve the three classification problems. The linear kernel was first selected to linearly classify the training set due to the significantly larger feature size than the sample size (27). Since our data are linearly non-separable, slack variables were employed during the training process to improve the generalization ability of the model by allowing some sample points to be misclassified. Additionally, the decision hyperplane was determined by soft margin maximization and dual problem settlement. The application of multiclass classification utilized a one-*vs*-rest voting strategy, which means that three binary classifiers are trained (28). Finally, samples from the test set were predicted separately and the category with the highest probability was subsequently assigned as the final prediction.

The XGBoost model contains *K* base learners in which each learner predicts the *X_i_* outcome of the *i*-th input and then acquires the final classification result by pooling each output *f_K_ (X_i_)* (11). The xgb.XGBClassifier function was adopted to build the model based on a set of relevant parameters such as learning rate, number of trees, and gamma. The grid search strategy was applied from the Sklearn interface to obtain the best-optimized hyperparameters, which optimizes the model’s performance and avoids overfitting issues (29). Next, the XGBoost model was trained using the determined parameters and 10-fold cross-validation (30). The most important features that were taken into consideration were as follows: distant organ metastasis, lymph node metastasis (including the number of lymph node metastases), HER-2, ER, PR, and Ki-67 expression; pathology grade; menopausal status; age; and lympho-vascular invasion. Eventually, values were predicted for the test set and evaluated by the module to obtain the reliability of the XGBoost model (31).

LSTM simulates the memory storage capacity of our brain, which develops novel artificial intelligence algorithms. Compared with traditional neural network algorithms, LSTM can precisely deal with more complex problems related to time series or sequential data (32, 33). In this study, the LSTM model was constructed in Keras. After learning meaningful features, dense layers were used to map features from the high-dimensional data space to a low-dimension representation space and finally become a column vector, in which the number of columns is the same as risk categories (34). Specifically, the first column corresponded to the low risk with “class 0,” the second column corresponded to the intermediate risk with “class 1,” and the third column corresponded to the high risk with “class 2.” Each patient would be obtained a column vector with a sum of 1 through the softmax_layer. For example, the predicted result for one patient was shown [0.2,0.7,0.1]. A predicted value of 0.2 represented the probability of class “0,” 0.7 represented the probability of class “1,” and 0.1 represented the probability of class “2.” This column vector indicated that this patient was finally classified as the maximum value of the predicted label “class-1” (intermediate-risk). Moreover, backpropagation was utilized to optimize the parameters of this model, thus minimizing the loss function (35). Feature units were randomly dropped through dropout layers during each feedforward training to avoid overfitting issues and obtain a generalization model.

To determine the favorable model, the performance of each model was compared through the receiver operating characteristic curve (ROC) and the area under the curve (AUC). Since our dataset has an imbalanced distribution of samples, consisting of disparate sample sizes in each class. Precision (positive predictive value)–recall (sensitivity) curves were also applied as indicators to further assess each model’s performance (36). Other important metrics for evaluation include accuracy, F1 score, macro-average, micro-average, and weight-average. A further explanation of these indicators is provided in the **Supplement Data**.

### Statistical analysis in patients

We divided the 85 patients treated with chemotherapy alone after surgery into chemo-sensitive and chemo-resistant groups based on each patient’s response to chemotherapy. The inclusion criteria of chemotherapeutic resistance are as follows (37, 38): (1) An increase in tumor volume after postoperative chemotherapy was observed using B-ultrasound and MRI; (2) sustained increases in tumor marker levels and clinical symptoms did not relieve; (3) and patients were confirmed as having progressive disease (PD) according to Response Evaluation Criteria in Solid Tumors (RECIST version 1.1). Chemotherapy resistance is considered when one or all of the criteria are met. In order to retrospectively validate the predictive effectiveness of our model, we next used a binary logistic regression approach with chemotherapy resistance as the dependent variable and the risk categories predicted by our model as the covariate (39).

For patients treated with neoadjuvant chemotherapy, the endpoint was time to progression (TTP) because a death event was not observed at the cutoff in this study. TTP was defined as the date from registration to invalid treatment or disease progression (40, 41). For subgroups only undergoing postoperative chemotherapy, the endpoint of interest was set as invasive disease-free survival (iDFS). iDFS is calculated as the time interval from the date of registration to the first recurrence of breast cancer, the development of contralateral primary breast cancer, or death from any cause (42).

Kaplan–Meier analysis and the log-rank test were used to assess survival outcomes in groups treated with neoadjuvant and postoperative chemotherapy as well as postoperative chemotherapy alone. All statistical analyses were implemented with the R software 3.5.0 (https://www.r-project.org/); a *P* value <0.05 was considered statistically significant.

## Results

### Training and test cohorts conducted

The included cohorts were randomly divided into training and test cohorts according to the ratio of 7(n = 1,289):3(n = 552) (**Table 2**) (43, 44). The validation set was considered a part of the training cohort to fine-tune the hyperparameters in our models. Each group of information was evenly distributed without bias. **Table 2** presents the characteristics of patients. Valuable information in EHRs was first segmented and annotated, including integrated pathological and clinical information from encounter notes and progress notes. Text snippets were further processed using feature extraction methods to extract specific string fields (45). The extractor achieved 95% accuracy, and each string was then matched against the numeric label “0” or “1”; all matched features of each patient were aggregated together to form a large dataset, which simplifies the learning process. This transformation process involved converting complex multiple input variables into a more manageable format, which greatly improved the classification performance of our model (46). The standards for automatic extraction are shown in the methods.

**Table 2:** Cohort characteristics for 30% train/70% test experiments in breast cancer patients.

Characteristic	Training set	Test set
Number of patients	1,289	552
Gender, %Female	1,282 (99.5)	549 (99.5)
Gender, %Male	7 (0.5)	3 (0.5)
Age, no. (%)		
<35	12 (0.9)	8 (1.4)
≥35	1,277 (99.1)	544 (98.6)
Menopausal status		
Pre	298	109
Post	984	440
Molecular subtypes		
Luminal A/luminal B	609	248
HER2+	429	178
Triple negative	251	126
Histology		
Invasive ductal carcinoma	1,067	443
Invasive lobular carcinoma	53	23
Mixed (IDC and ILC)	48	16
DCIS/LCIS	86	58
Other types	35	12
Recurrence risk assessment		
Low-risk	102	86
Intermediate-risk	758	283
High-risk	429	183

IDC, invasive ductal carcinoma; ILC, invasive lobular carcinoma; DCIS, ductal carcinoma in situ; LCIS, lobular carcinoma in situ; F, female; HER2, human epithelial growth factor receptor-2.

### SVM, XGBoost, and LSTM models predicted the recurrence risk of postoperative breast cancer patients

**Figure 2:**
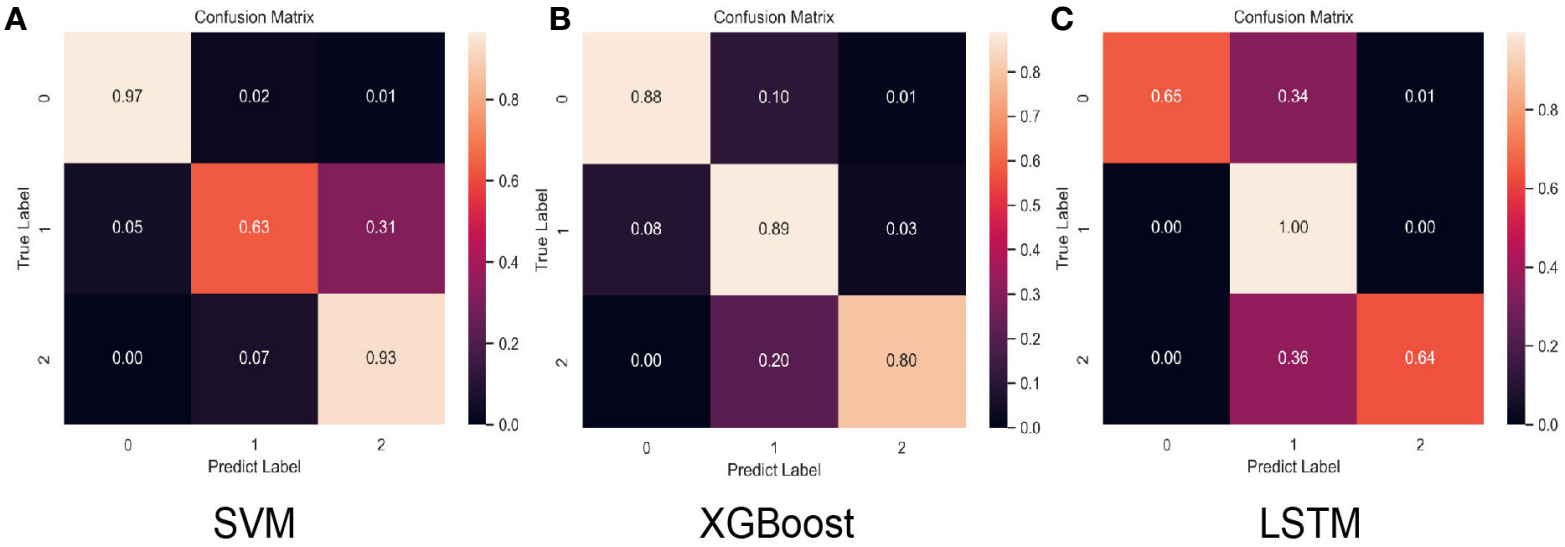
Normalized confusion matrix for the test set of each model. “Class 0,” “class 1,” and “class 2” correspond to low-risk, intermediate-risk, and high-risk categories. **(A)** SVM confusion matrix, **(B)** XGBoost confusion matrix, **(C)** LSTM confusion matrix.

**Figure 3:**
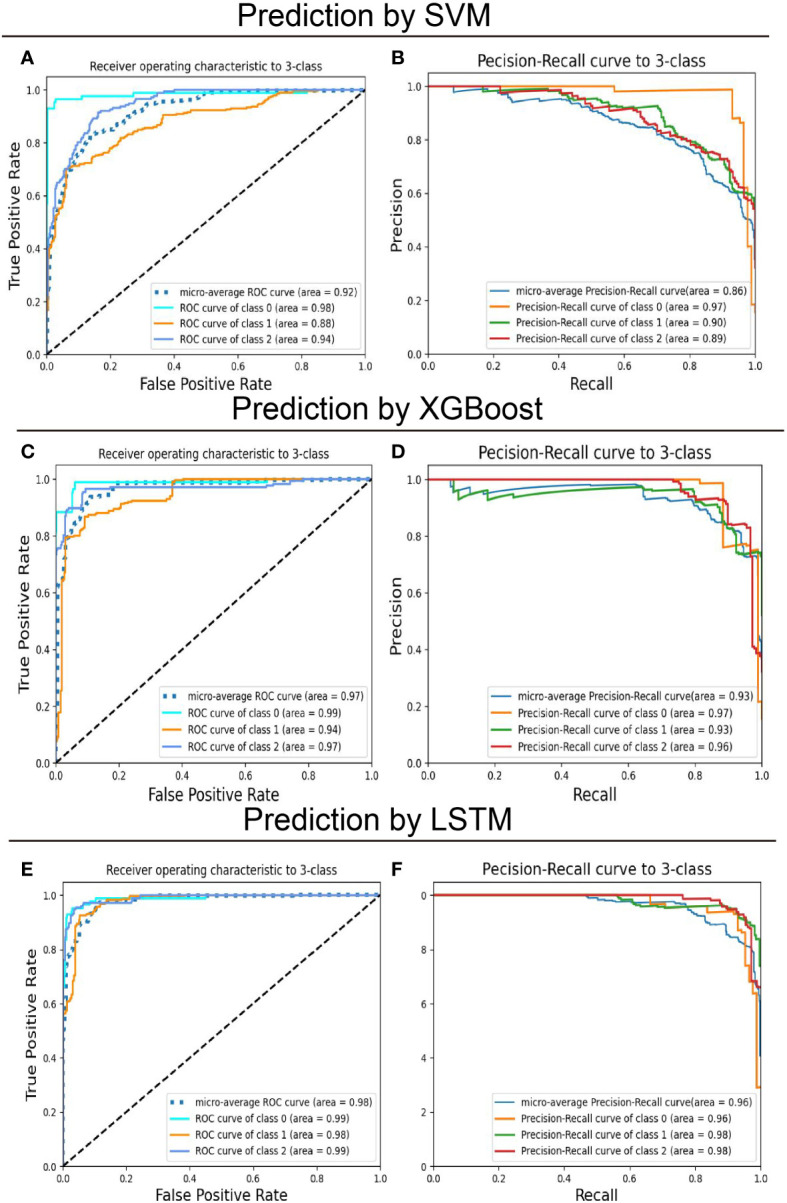
Predictive performance of models on the training set for multiclassification of breast cancer patients. The support vector machines (SVM), extreme gradient boosting (XGBoost), and long short-term memory (LSTM) recurrent neural network models were trained to classify patients with operated breast cancer from the feature label values. **(A, C, E)** Receiver-operating characteristics (ROC) curve and **(B, D, F)** Precision-recall (PR) curve for the test set was shown to quantify the performance of models. “Class 0,” “class 1,” and “class 2” correspond to low-risk, intermediate-risk, and high-risk categories.

After model development with the training subset, test samples were uploaded to predict recurrence risk, and this multi-classification task was conducted *via* a one-*vs*-the-rest method. Specifically, when one category was correctly predicted by the model, the remaining categories were considered negative (47), thus generating a confusion matrix for each category (**Figure 2**). We computed the evaluation metrics of each category based on the confusion matrix, such as accuracy, precision, recall, and the area under the receiver-operating characteristic curve (ROC-AUC) (**Table 3** and **Figure 3**). In order to further compare the effectiveness of models, we averaged (macro-average, F1 score) and weighted (micro-average, weighted-average) the evaluation indicators of each category (**Table 3**) (47, 48). Subsequently, we draw the ROC curve for each prediction category with the true positive rate (TPR) as the abscissa and the false positive rate (FPR) as the ordinate and explained the achievement of each model using a micro-average ROC curve (**Figures 3A, C, E**). The AUC values of the micro-average ROC curve corresponding to SVM, XGBoost, and LSTM were 0.92 ± 0.06, 0.97 ± 0.03, and 0.98 ± 0.01 (**Figures 3A, C, E**). Additionally, the area under the precision–recall curve (AUC-PR) is more suitable for assessing performance metrics on processing imbalanced data compared with the area under the receiver operating characteristic curve (AUC-ROC) (49–51). The SVM generated the smallest micro-average AUC-PR (0.86 ± 0.11), and the LSTM model demonstrated the largest micro-average AUC-PR (0.96 ± 0.02), which indicates that a great number of patients were correctly labeled (**Figures 3B, D, F**). Overall, the LSTM model accomplished superior performance on the test set, with a micro-averaged AUC-PR that represents an improvement of 10% and 3% compared with SVM and XGBoost. The LSTM model manifested a significantly higher accuracy (0.89), F1 score, macro-F1 score (0.87), and weighted-F1 (0.89) (**Table 3**).

**Table 3:** Comparison of test set prediction performance between the models.

	Precision	Recall	F1 score	Accuracy
SVM				
Low-risk	0.85	0.97	0.90	0.78
Intermediate-risk	0.93	0.63	0.75	
High-risk	0.64	0.93	0.76	
Macro avg	0.81	0.84	0.81	
Weighted avg	0.82	0.78	0.78	
XGBoost				
Low-risk	0.76	0.88	0.82	0.86
Intermediate-risk	0.85	0.89	0.87	
High-risk	0.94	0.80	0.86	
Macro avg	0.85	0.86	0.85	
Weighted avg	0.86	0.86	0.86	
LSTM				
Low-risk	1	0.65	0.79	0.89
Intermediate-risk	0.83	1	0.91	
High-risk	0.99	0.84	0.91	
Macro avg	0.94	0.83	0.87	
Weighted avg	0.91	0.89	0.89	

### Breast cancer patients at high recurrence risk are more likely to be resistant to chemotherapy after surgery

**Figure 4:**
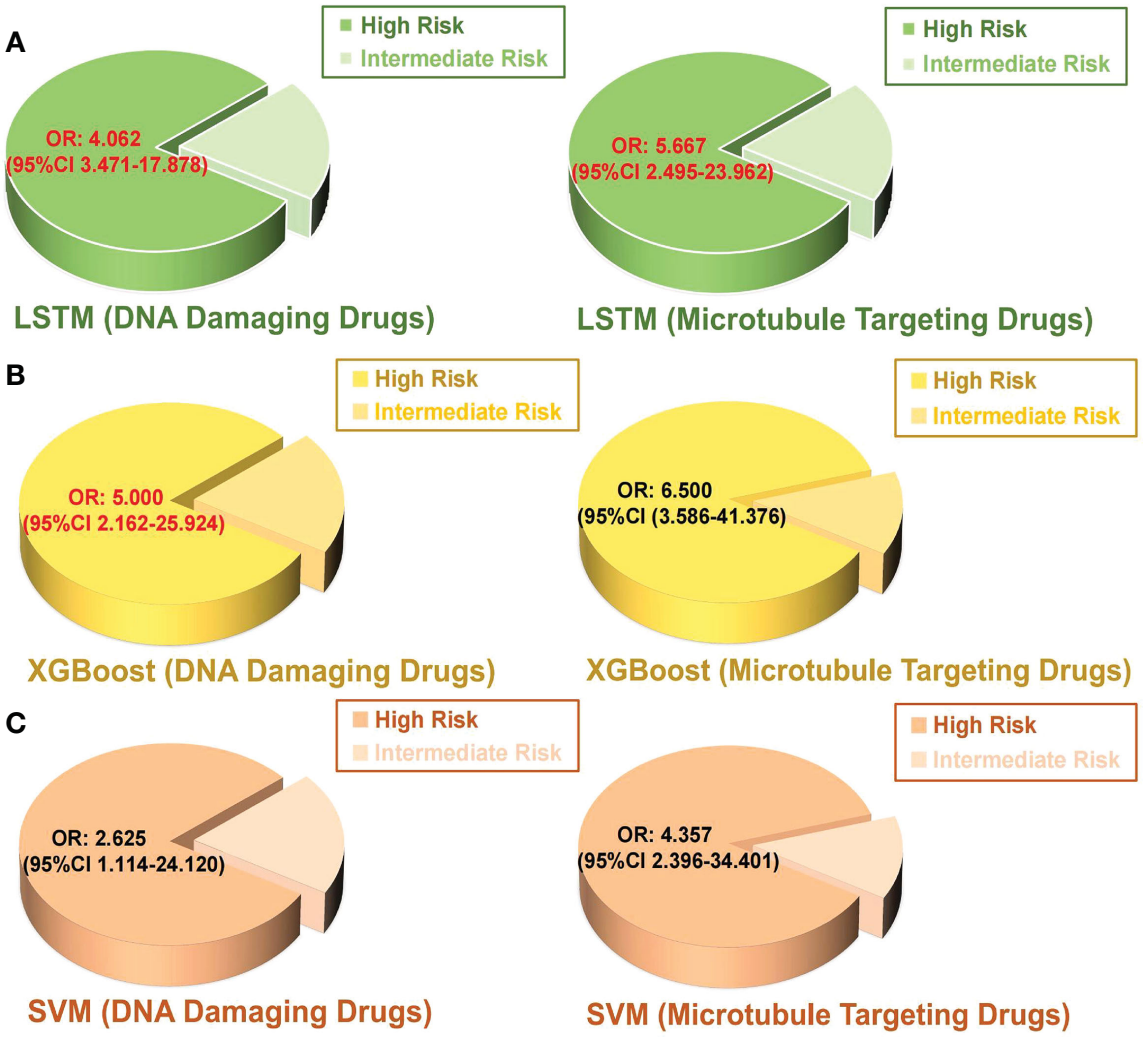
Binary logistic regression was performed to analyze the relationship between the predicted intermediate risk and high risk by each model and chemotherapy resistance in postoperative breast cancer patients. **(A)** LSTM, **(B)** XGBoost, **(C)** SVM. OR value: odd ratio; The red color indicates a statistically significant correlation *P* < 0.05.

Chemotherapy resistance is the most crucial reason for recurrence of breast cancer patients after surgery (52). In order to exclude the influence of other treatment options on the effect of chemotherapy, patients who received chemotherapy alone were included in the experiment. A binary logistic regression analysis was executed to identify the association between model-based predicted recurrence risk and chemotherapy resistance in breast cancer. The inclusion criteria for chemotherapy resistance in this study are described in the methods. A total of 432 patients received postoperative treatment, and 85 (20%) patients underwent chemotherapy alone, which included DNA-damaging drugs such as anthracyclines and platinum and microtubule-targeting drugs like paclitaxel. There were 37 patients classified as high-risk by the LSTM model, 32 of which (86%) were chemotherapy resistant. Among the 46 intermediate-risk patients predicted by the LSTM model, 29 (63%) patients were chemotherapy resistant (**Table 4**). The results of binary logistic regression showed that the probability of DNA-damaging drug resistance in high-risk patients predicted by the LSTM model was 4.062 times more than in intermediate-risk patients (*P* < 0.05; **Figure 4A**). Meanwhile, the high-risk patients predicted by the LSTM model were more likely to be resistant to microtubule-targeted drugs than the intermediate-risk patients (high-risk: intermediate-risk = 5.667: 1; *P* < 0.05; **Figure 4A**). These results suggest that high-risk patients predicted by our model are more resistant to chemotherapy drugs after surgery and likely to perform more insensitively to paclitaxel. Consistent results were observed in the SVM and XGBoost models, but the *P* values are not significant (**Figures 4B, C**). We did not include the low-risk patients because the number of low-risk samples was insufficient to meet the minimum sample size (n = 10) required for binary logistic regression analysis.

**Table 4:** Predictive performance of the LSTM model for postoperative breast cancer patients treated with chemotherapy alone.

Number of patients	Recurrence risk assessment Low-risk (AUC ± SD)	Recurrence risk assessment Intermediate-risk (AUC ± SD)	Recurrence risk assessment High-risk (AUC ± SD)
Chemo-sensitive	2 (0.92 ± 0.03)	17 (0.87 ± 0.04)	5 (0.85 ± 0.08)
Chemo-resistant	0	29 (0.84 ± 0.07)	32 (0.86 ± 0.11)

The model’s performance was assessed through the area under the curve (AUC) ± standard deviation (SD).

### Our model can predict the neoadjuvant chemotherapy benefits and the survival of patients

Neoadjuvant therapy plays an important role in the clinical practice of systemic treatment for breast cancer patients (53). Nevertheless, recent research has reported that neoadjuvant chemotherapy is not necessarily beneficial for patient survival. Patients who were refractory to neoadjuvant treatment can result in a higher local recurrence rate after surgery (54, 55). Among our subgroups treated with neoadjuvant chemotherapy, 52 and 72 patients were predicted to be intermediate and high risk by LSTM, respectively (**Table 5**). Contrary to our anticipated outcome, the results indicate that the majority of patients who received neoadjuvant chemotherapy did not exhibit a low-risk profile as we had expected. Moreover, 43 and 23 patients treated with the postoperative chemotherapy alone were predicted as intermediate risk and high risk by LSTM. These results indicated that not all breast cancer patients should receive neoadjuvant chemotherapy before surgery. Our predictive model can be utilized to evaluate the benefit of patients receiving neoadjuvant chemotherapy.

**Table 5:** Predictive performance of the LSTM model for breast cancer patients treated with neoadjuvant and postoperative chemotherapy or postoperative chemotherapy alone.

Number of patients	Recurrence risk assessment Low-risk (AUC ± SD)	Recurrence risk assessment Intermediate-risk (AUC ± SD)	Recurrence risk assessment High-risk (AUC ± SD)
Neoadjuvant and postoperative chemotherapy	3 (0.93 ± 0.01)	52 (0.91 ± 0.03)	72 (0.95 ± 0.03)
Postoperative chemotherapy alone	2 (0.89 ± 0.04)	43 (0.87 ± 0.02)	23 (0.89 ± 0.01)

For patients treated with neoadjuvant and postoperative chemotherapy or postoperative chemotherapy alone, the model was trained to extract postoperative information to classify “high-risk,” “intermediate-risk,” and “low-risk” labels. The model’s performance was assessed through the area under the curve (AUC) ± standard deviation (SD).

**Figure 5:**
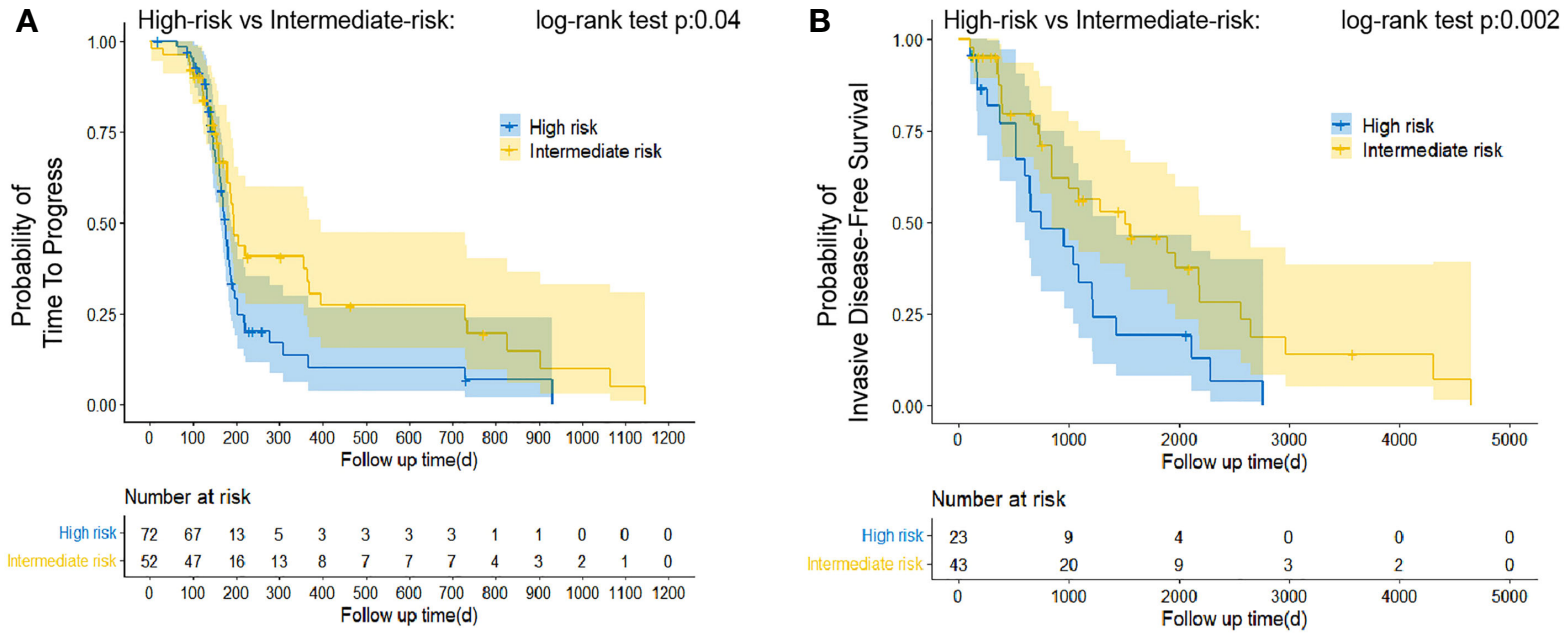
Estimation of relative survival in classified patients treated with neoadjuvant therapy and postoperative chemotherapy alone by Kaplan–Meier curve analysis. Patients predicted to be classified as “Intermediate-risk” presented favorable TTP **(A)** and iDFS **(B)** than that patient identified as “high-risk.” The log-rank test was appropriate to assess performance.

Data were next manually extracted on time to disease progression (TTP), which was considered a reliable surrogate endpoint in advanced cancer with medical therapy (Lee, Jang, Lee, Cho, Lee, Yu, Kim, Yoon, Kim, Han, Oh, Im and Kim 2016). For patients administered neoadjuvant chemotherapy, the intermediate-risk operated patients predicted by the LSTM model was shown to have a longer TTP than the high-risk ones (*P* < 0.05; **Figure 5A**). We compared invasive disease-free survival (IDFS) in the groups that received only postoperative chemotherapy and found that the high-risk patients acquired poorer IDFS than the intermediate-risk ones (*P* < 0.05; **Figure 5B**). Compared with intermediate-risk or high-risk, the low-risk sample size was insufficient to create reliable estimates. However, low-risk patients actually had better outcomes according to their clinical information. Therefore, our model can accurately predict the prognosis of breast cancer patients before treatment and suggest that clinicians provide the most appropriate treatment regimen for patients, such as whether to administrate patients with neoadjuvant chemotherapy or postoperative chemotherapy.

## Discussion

In this study, the advantages and limitations of our proposed model are as follows: (i) All models can seamlessly classify from labeled data with an accuracy of over 75%. (ii) The linear SVM model generates a good non-linear mapping between input and output variables. It has good robustness and appears to have no effect on the model when non-supported vector samples are added and removed, thus avoiding the problems of leaf node selection in XGBoost and dimension disaster in LSTM. (iii) The XGBoost model excited more parameters and performed more accurately than SVM. It illustrated a white box compared with ANN so that the model’s effectiveness can be intuitively evaluated. Moreover, the XGBoost model has presorted features based on the parameters before training, which were repeatedly utilized in subsequent iterations, significantly reducing the computation. (iv) LSTM realized the highest accuracy among all models, attributed to the continuous optimization of gradient descent and backpropagation. (v) The high recurrence risk predicted by the LSTM model was consistent with the chemotherapy resistance and the worse prognosis of postoperative patients, which corresponded to the actual situation. (vi) The SVM algorithm is less sensitive to the handling of missing data. Clearly, vacant values were filled with the default value “0” during data preprocessing, which affects the linear separability in the feature space of SVM. Nevertheless, the XGBoost algorithm tries different methods at each node and identifies the best method to handle when missing data are encountered. LSTMs can learn complex correlations between features, including further details in default values. (vii) The model uses only a single type of input information that converts textual clinical reports into labeled values. Once new variables emerge, we will manually develop and validate a new set of regular expressions for each specific task.

We established machine learning algorithms capable of extracting patient classification information from unstructured clinical notes. Benefitting from the application of technologies and frameworks of machine learning, our models for screening diagnostics with low-cost burden were favorable (56). However, several unavoidable challenges with machine learning were posed. First, the data annotation and processing were complicated. In order to achieve data collection and annotation with high precision, including the term standardization of biological features, the variability of descriptive words, and the presence of negative phrases, we searched for each key term and encoded it with category encoders through feature engineering and natural language processing. High accuracy was achieved eventually for each feature of information abstracting. Secondly, for high-dimensional scene data exploration (such as medical time-series data), the XGBoost algorithm cannot effectively eliminate noise variables (57). Therefore, we conducted a grid search to determine the algorithms of optimal dimensionality reduction and added randomness to improve robustness (58). Additionally, an increasing fraction of the training time in the LSTM model would reduce the number of iterations within the same total training time (59). We utilized forward calculation and backpropagation to continuously adjust the parameters for extracting the optimal features. Therefore, we provided a reproducible predicted tool to predict the recurrence risk of breast cancer patients after surgery.

To further guide clinical practice, our models maintained their performance in reflecting patient tolerance to chemotherapy drugs. We verified that high-risk patients tend to be more resistant to DNA damage and microtubule inhibition drugs than intermediate-risk patients. This result provides a basis for the clinical treatment application of different drugs to postoperative breast cancer patients. Chemotherapy resistance is not only an important risk factor for cancer recurrence but also a major cause of poor patient outcomes (52). Meanwhile, our models also validated the prognosis of patients who underwent neoadjuvant chemotherapy and postoperative chemotherapy. Since the linkages between EHR data and death registries were rare, we used TTP or IDFS as surrogate endpoints to assess differences in survival outcomes of predicted categories. Our approach highlighted the importance of estimating the recurrence risk after neoadjuvant chemotherapy, indicating whether patients routinely receive preoperative chemotherapy is worth thought-provoking (60, 61). Although patients classified as low-risk were predicted in our model, the recurrence was not statistically significant compared with the other two groups because of the rare number of samples.

Previous studies have applied natural language processing to abstract biological factors from medical records to predict breast cancer staging based on the American Joint Committee on Cancer (AJCC) staging manual (62). In 2020, researchers also implemented artificial neural networks to predict breast cancer prognosis by selecting crucial survival factors, including tumor size, tumor staging, lymph node metastasis, and other related variables (63). Moreover, deep learning has shown promise in predicting breast cancer risk rates by extracting factors such as age, race, and menstrual history (64). In contrast, our approach significantly solved the bottleneck of extracting outcomes from a great number of clinical texts and achieved effective feature extraction in different scenes. Additionally, those included studies were predominantly conducted in the United States or Europe, but the data for Asian breast cancer patients remained unknown. Breast cancer incidence is strongly correlated with variations in geographic distribution (65, 66). Because of differences in people’s diets and lifestyles, breast cancer is highly prevalent in the alpine region (67), such as the northeast of China. An accurate assessment of patients’ recurrence risk before tailored individual treatment plans can provide valuable guidance on improving patient outcomes. Our studies contribute to the development of screening strategies for breast cancer in the Asian population.

In conclusion, we developed AI-based models that integrate histopathological features of breast cancer and clinical information from preprocessed clinical notes to predict the recurrence risk of postoperative breast cancer patients. The performance and generalizability of our model have emphasized the potential application in the estimation of recurrence risk in breast cancer patients.

## Data availability statement

The raw data supporting the conclusions of this article will be made available by the authors, without undue reservation.

## Ethics statement

The studies involving human participants were reviewed and approved by Ethics Committee of Harbin Medical University. The patients/participants provided their written informed consent to participate in this study. Written informed consent was obtained from the individual(s) for the publication of any potentially identifiable images or data included in this article.

## Author contributions

WY offered main direction and significant guidance of this manuscript. LZ, LL, DC and HL drafted the manuscript and illustrated the figures for the manuscript. LL provided the clinical data. YX and HB helped with the data analyzed. All authors contributed to the article and approved the submitted version.
