# Supplementary material for: The innovative model based on artificial intelligence algorithms to predict recurrence risk of patients with postoperative breast cancer
Source: Front Oncol. 2023 Mar 7;13:1117420. doi: 10.3389/fonc.2023.1117420 (PMC10029918; doi:10.3389/fonc.2023.1117420)
Supplement: Supplementary file 1 [file DataSheet_1.docx]

SUPPLEMENTARY CONTENT

**The Innovative Model Based on Artificial Intelligence Algorithms to Predict Recurrence Risk of Patients with Postoperative Breast Cancer**

**Contents:**

**Supplemental Methods**

**Supplemental Tables**

Supplemental Table 1. Cohort Characteristics (n=1841).

Supplemental Table 2. Confusion matrix for metric extractors (1841 snippets).

Supplemental Table 3. Risk categories for operated breast cancer patients according to the CACA-CBCS (Chinese Anti-Cancer Association, Committee of Breast Cancer Society) clinical guidelines for the diagnosis and treatment of Breast cancer, 2021.

Supplemental Table 4. Standardization descriptions for tumor size.

**Supplemental Methods**

**Rules for standardizing data.**

1. The lesion size was measured in millimeters, such as "18 × 19 mm in size". Unit values of all sizes were converted into specific units (e.g., millimeter to centimeter).
2. To value the primary tumor size, descriptions for different sizes such as "egg yolk", "peanut rice", "fist" and "fava bean ", etc., all of these would be converted into standardized values to represent (Supplementary Table **4**).
3. To validate the pathology grade of breast cancer, descriptions for different forms such as "Grade 1", "grade Ⅰ", "Well-differentiated" and "Low-grade ", all of these would be considered “G1= Ⅰ” as values for representing the output.
4. To extract the status of estrogen receptor (ER) and progesterone receptor (PR) in breast cancer, we analyzed information from validated immunohistochemical reports. ER (+) was classified as "estrogen receptors are present in breast cancer cells "; else, it was "ER (-)". Similarly, PR (+) was classified as "progesterone hormone receptors are present in breast cancer cells"; else, it was "PR (-)". Positive for ER or PR if ≥1% of the tumor cell nuclei were immunoreactive. The function was shown output in the form of "(+) =1" and "(-) =0".
5. To extract the status of HER2/cerB-2 receptor (human epidermal growth factor receptor 2) in breast cancer, we analyzed information from validated reports of immunohistochemistry or fluorescence in situ hybridization (FISH). score 0 from score 1+ of immunohistochemistry in HER2 was classified as "HER2 negative", which shown that HER2/neu gene neither overexpressed nor amplified. A score of 2+ is borderline; positive for HER2 if FISH had been detected gene amplification; else, it was "negative for HER2". score 3+ of immunohistochemistry in HER2 was classified as "HER2 positive". The function was shown output in the form of "positive =1" and "negative =0".
6. Lymphovascular invasion (+) was classified as " the presence of tumor cells into lymphatic or blood vessels "; else, it was "Lymphovascular invasion (-)". The function was shown output in the form of "(+) =1" and "(-) =0".
7. We also converted numbers in textual form, to numerical values. Most descriptions were of the following forms:

**7.1.** "The number of lymph node metastases is 3/14" would be extracted to obtain the values of metastatic lymph nodes.

**7.2.** Percentage of Ki-67 such as "15%" would be extracted to obtain the values.

Moreover, in order to standardize the different representations of semantically similar concepts, we added additional criteria by searching the Unified Medical Language System (UMLS) (Humphreys et al. 2020, Lindberg et al. 1993). For instance, "lymph node metastasis" was described in various ways, like "axillary metastasis in cancer", "cancer infiltrating subclavian region", "LN 1/10" and "positive sentinel lymph node". We also focused on details such as abbreviations (e.g., "IDC", "DCIS"), grammatical errors, and capitalization.

**Definition of evaluation metrics.**

**1.** Accuracy: The measure that computes each closeness of the prediction to actual values.

**2.** Precision: The ability of a classification model to identify true positives among all the predicted positives. Mathematically, precision is the number of true positives divided by the number of true positives plus the number of false positives.

**3.** Recall: The ability of a model to find all the positive cases. Mathematically, we defined recall as the number of true positives divided by the number of true positives plus the number of false negatives.

**4.**F1 score: The evaluation metric for a classification defined as the harmonic mean of precision and recall. Mathematically, it was expressed as follows:


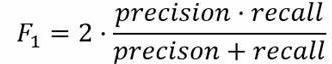


*“****·****”* **denotes** the multiplication of values.

**5.** Macro avg F1 score: The arithmetic means of the individual class related to precision, recall, and F1 score. We used macro average scores when all classes were needed to treat equally to evaluate the overall performance of the classification model against the most common class labels. In our models, the Macro average F1 score represented the arithmetic mean between the F1 scores of the three categories.

**6.** Micro average: Micro-average will aggregate the contributions of all classes to compute the average metric, which takes imbalance into account. In our models, the micro average precision was**the sum of true positives for a single class divided by the sum of predicted positives for all classes.** The micro average recall score was the sum of true positives for a single class divided by the sum of true positives for all classes.

**7.**Weighted avg F1 score: Weighted average is considered the average where a weight is assigned to each of the quantities that are needed to be averaged. In our models, we added the F1 score together for each class by using a weight that depends on the number of true labels of each class. Mathematically, it was expressed as follows:


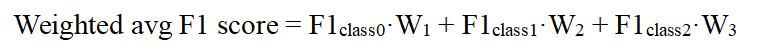


*“****·****”* **denotes** the multiplication of values.

**Supplemental Tables**

**Supplemental Table 1**. **Cohort Characteristics (n=1841).**

| **Characteristic** |  | **Number Of Patients (n=)** |
| --- | --- | --- |
| Age, mean 59 years  (Range, 24-93) | <35 yr | 20 |
|  | ≥35 yr | 1821 |
| Gender | Female | 1831 |
|  | Male | 10 |
| Menopausal Status | Premenopause | 407 |
|  | Postmenopause | 1424 |
| Molecular Subtypes | Luminal A | 417 |
|  | Luminal B | 440 |
|  | HER2+ | 607 |
|  | Triple negative | 377 |
| Histology | Invasive Ductal Carcinoma | 1510 |
|  | Invasive Lobular Carcinoma | 76 |
|  | Mixed (IDC and ILC) | 64 |
|  | DCIS/LCIS | 144 |
|  | Other types | 47 |
| Neoadjuvant Chemotherapy | Yes | 140 |
|  | No/unknown | 1701 |
| Postoperative Chemotherapy | Yes | 369 |
|  | No/unknown | 1472 |
| Patients Followed after Surgery | Recurrence at Follow-up | 905 |
|  | Metastasis at Follow-up | 532 |
|  | Death at Follow-up | 3 |

Abbreviations: IDC: invasive ductal carcinoma; ILC: invasive lobular carcinoma; DCIS: ductal carcinoma in situ; LCIS: lobular carcinoma in situ; HER2: human epithelial growth factor receptor-2.

**Supplemental Table 2. Confusion matrix for metric extractors (1841 snippets).**

| **Actual** | | |
| --- | --- | --- |
| **Predicted** | 1553^TP^ | 38^FP^ |
|  | 49^FN^ | 201^TN^ |

The accuracy can be calculated as *(TP + TN)/ (TP + TN + FP + FN)*.

**Supplemental Table 3**. **Risk categories for operated breast cancer patients according to CACA-CBCS (Chinese Anti-Cancer Association, Committee of Breast Cancer Society) clinical guidelines for the diagnosis and treatment of Breast cancer, 2021 (**Breast Cancer Expert Committee of China Anti-Cancer 2021**).**

| **Risk category** | | | |
| --- | --- | --- | --- |
| **Low risk** | Node negative | ①All of the following features: | pT ≤ 2 cm |
|  |  |  | Grade Ⅰ |
|  |  |  | Absence of lympho-vascular invasion |
|  |  |  | HER2/neu gene neither overexpressed nor amplified |
|  |  |  | Estrogen receptor-positive OR Progesterone receptor-positive |
|  |  |  | The median pathology Ki-67 ≤ 20% |
|  |  |  | Age ≥ 35 years |
|  |  | ②Estrogen receptor-positive AND HER2/neu gene neither overexpressed nor amplified AND Multi-gene panel testing: Low risk | |
| **Intermediate risk** | Other conditions that not be presented in low risk OR high risk | | |
| **High risk** | Node positive (1–3 involved nodes) AND at least one of the following features: | ①Estrogen receptor-positive OR Progesterone receptor-positive AND HER2/neu gene neither overexpressed nor amplified, AND at least one of the following features: | pT > 5 cm |
|  |  |  | Grade Ⅲ |
|  |  |  | Multi-gene panel testing: High risk |
|  |  | ②Estrogen receptor-negative AND Progesterone receptor-negative OR  HER2/neu gene overexpressed or amplified | |
|  | Node positive (4 or more involved nodes) | | |

The framework is grounded on the 2005 St Gallen consensus statement and refined in application of actual situation in China (Goldhirsch et al. 2005). pT: pathological tumor size; Grade: histologic grade/nuclear grade; HER-2/neu must be determined by immunohistochemical fluorescence (IHC), fluorescence in situ hybridization (FISH), or chromogenic in situ hybridization (CISH) with strict quality control assessment, IHC and FISH indicators are used as data sources in this paper.

**Supplemental Table 4**. **Standardization descriptions for tumor size.**

| **Tumor Size** | **pT ≤ 2cm** | "Peanut rice"/"a grain of rice"/"a grain of soya beans" /"the thumbnail" | |
| --- | --- | --- | --- |
|  | **pT ＞2cm** | **pT＜5cm** | "coin"/"egg yolk"/"egg"/"peanut"/"pigeon egg" |
|  |  | **pT ≥ 5cm** | "Goose egg"/"apple"/"duck egg"/"orange"/"cobblestone"/"fist" |

Based on the writing style of medical experts, we explained the corresponding terms of tumor size with descriptions. For example, the size of the "fist" shown in the medical records will be considered "tumor size ≥5cm" as values for representing the output.

**References**

Breast Cancer Expert Committee of China Anti-Cancer A. 2021. [Guidelines for clinical diagnosis and treatment of breast cancer in China (2021 Edition)]. Zhonghua Zhong Liu Za Zhi.31:954-1040.

Goldhirsch A, Glick JH, Gelber RD, Coates AS, Thurlimann B, Senn HJ, Panel m. 2005. Meeting highlights: international expert consensus on the primary therapy of early breast cancer 2005. Ann Oncol. Oct;16:1569-1583. Epub 2005/09/09.

Humphreys BL, Del Fiol G, Xu H. 2020. The UMLS knowledge sources at 30: indispensable to current research and applications in biomedical informatics. J Am Med Inform Assoc. Oct 1;27:1499-1501. Epub 2020/10/16.

Lindberg DA, Humphreys BL, McCray AT. 1993. The Unified Medical Language System. Yearb Med Inform.41-51. Epub 1993/01/01.
